# Clinical Development of PARP Inhibitors in Treating Metastatic Castration-Resistant Prostate Cancer

**DOI:** 10.3390/cells8080860

**Published:** 2019-08-09

**Authors:** Jacob J. Adashek, Rohit K. Jain, Jingsong Zhang

**Affiliations:** 1 Department of Internal Medicine, University of South Florida, H. Lee Moffitt Cancer Center & Research Institute, Tampa, FL 33612, USA; 2 Department of Genitourinary Oncology, H. Lee Moffitt Cancer Center & Research Institute, 12902 USF Magnolia Drive, Tampa, FL 33612, USA

**Keywords:** PARP inhibitors, DNA damage repair deficiency, prostate cancer, targeted therapy

## Abstract

The approval of upfront abiraterone for castration-sensitive prostate cancer and the approval of enzalutamide and apalutamide for non-metastatic castration-resistant prostate cancer have led to early utilization of potent androgen receptor (AR) signaling inhibitors in treating advanced prostate cancer. There is an unmet need to develop novel therapies beyond targeting AR signaling for metastatic castration-resistant prostate cancer (mCRPC). Poly (ADP-ribose) polymerase inhibitors (PARPi) belong to a class of targeted agents being developed for the treatment of homologous recombination repair (HRR) deficient tumors. Olaparib, rucaparib, niraparib, veliparib, and talazoparib were evaluated in early phase trials as a monotherapy for HRR-deficient mCRPC. Among them, olaparib and rucaparib have breakthrough designations for *BRCA1/2*-mutated mCRPC. Phase II studies also reported clinical activity of the PARPi and abiraterone combination and the PARPi checkpoint inhibitor combination in HRR-intact mCRPC. Ongoing phase III trials are testing these combinations as frontline or later line treatments for mCRPC. This review summarizes the critical clinical data as well as ongoing clinical trials for developing PARPi in treating mCRPC.

## 1. Introduction

The intricacies of the genomic landscape of prostate cancer are continuously being unraveled with increased abilities to sequence the tumor DNA, and cell free circulating tumor DNA. Analysis of pooled next-generation sequencing (NGS) data involving a total of 962 prostate cancer patients reported 11.8% of metastatic prostate cancer patients and 4.6% of localized prostate cancer patients carried germline deleterious mutations in DNA damage repair (DDR) genes [1]. Among these DDR genes, the majority of them were involved in DNA homologous recombination repair (HRR) with *BRCA2* (5.3%) being the most common mutated gene, followed by *CHEK2* (1.9%), *ATM* (1.6%), and *BRCA1* (1%). Cells compensate for deleterious mutations in *BRCA1* or *BRCA2* by upregulating the poly (ADP-ribose) polymerase (PARP) enzyme complex to continue the cell cycle [2]. PARP binds directly to single-strand DNA breaks, catalyzes the cleavage of nicotinomide adenine dinucleotide-plus into nicotinamide and ADP-ribose, poly (ADP-ribosyl) ation, then occurs through attaching several ADP-ribose to the target protein. The PARP family is currently comprised of 18 members. PARP1 and PARP2 are known to be involved in DNA damage repair. PARP1 knock-out mice display no phenotypic abnormalities. Cells lacking PARP1 show normal apoptotic responses to treatment with antibodies against FAS, TNF-alpha, gamma-irradiation, and dexamethasone, indicating that PARP1 is dispensable in apoptosis. 

PARP inhibitors (PARPi) work at the DNA level during replication by forming a complex with the PARP1 and PARP2 enzymes. Inhibition of PARP with PARPi leads to unrepaired DNA breaks that are normally repaired by the HRR during cell cycle late S to G2 phase. Unlike non-homologous end joining, HRR requires a homologous DNA template and is considered a high-fidelity pathway to repair the double-strand DNA breaks. Cancer cells with deleterious mutations in *BRCA1* or *BRCA2* have defective HRR and the unrepaired DNA after treatment with PARPi will eventually lead to cancer cell death, a phenomenon called synthetic lethality. More recently, PARPi have been shown to trap the PARP1 and PARP2 enzymes at the damaged DNA. Trapped PARP-DNA complexes were more cytotoxic than unrepaired single-strand breaks caused by PARP inactivation [3].

Besides *BRCA1* [4], *BRCA2* [5], preclinical studies indicate that deleterious mutations in other genes in the HRR pathway might be associated with PARPi sensitivity. These include *ATM* [6], *ATR* [7], *CHK1* [7], *CHK2* [7], *DSS1* [7], *RPA1* [7], *NBS1* [7], *FANCD2* [7], *FANCA* [7], *FANCC* [7], *CDK12* [8], *PALB2* [9], *PTEN* [10], *RAD51C* [11], and *RAD54* [7]. In this review, we will discuss existing and emerging data on the development of PARPi in the treatment of advanced prostate cancer. 

## 2. Current Food and Drug Administration-Approved PARP Inhibitors

There are currently five PARPi being tested in clinical trials for various solid tumors (Table 1). Among them, olaparib was first approved in December 2014 for treating germline *BRCA*-mutated (gBRCAm) metastatic ovarian cancer after three or more lines of chemotherapy. This accelerated approval was based on an objective response rate (ORR) of 34% and a median duration of response (DOR) of 7.9 months in a single-arm phase II study involving 137 participants with heavily pre-treated gBRCAm ovarian cancer [12]. The BRACAnalysis CDx’s test was also approved as a companion diagnostic. Rucaparib was later approved in 2016 for the same disease setting with inclusion of somatic as well as germline *BRCA* mutations. 

In the setting of maintenance therapy, olaparib, niraparib, and rucaparib have been approved for adult patients with recurrent epithelial ovarian, fallopian tube, or primary peritoneal cancer who are in a complete or partial response to platinum-based chemotherapy. These approvals were based on improved progression-free survival (PFS) with PARPi maintenance therapy compared to placebo in randomized, double-blinded studies (study 19 [13], ARIEL3 [14], and NOVA [15]). Of note, testing for *BRCA* mutations is not required under these approvals. Response to platinum-based therapy was used as a biomarker to predict response to PARPi. Indeed, statistically significant improvement in PFS was observed in non-gBRCAm ovarian cancers in the phase III NOVA trial with niraparib, but the group with gBRCAm had the longest improvement in PFS followed by the group with homologous recombination repair deficiency (HRD) and non-gBRCAm ovarian cancer. Long-term follow-up on the olaparib maintenance study also reported improvement of overall survival (OS) compared to placebo irrespective of BRCA1/2 mutation status [16]. More recently, olaparib was approved in December 2018 as a maintenance treatment for patients with deleterious or suspected deleterious gBRCAm or somatic *BRCA*-mutated advanced epithelial ovarian, fallopian tube, or primary peritoneal cancer who were in complete or partial response to first-line platinum-based chemotherapy. Such approval was based on significant reduction in the risk of disease progression or death by 70% with olaparib maintenance therapy compared to placebo in the phase III SOLO-1 study (HR 0.30 (95% CI 0.23–0.41), *p* < 0.0001) [17].

Outside of ovarian cancer, olaparib and talazoparib were approved by the FDA to treat gBRCAm, HER2-negative, locally advanced, or metastatic breast cancer patients who have received treatment with an anthracycline and/or a taxane (unless contraindicated) in the neoadjuvant, adjuvant, and/or metastatic treatment setting. Both approvals were based on significant improvement of PFS when compared to physician’s choice of chemotherapy in the randomized OlympiAD (NCT02000622) and EMBRACA (NCT01945775) trials [18,19].

Although the PARPi under clinical development have distinct structures, different PARP trapping potency and half-lives (Table 1), cytopenias (particularly anemia), fatigue, and nausea are the most common side effects shared by this class of agents. Serious side effects like myelodysplastic syndrome/acute myeloid leukemia (<2%) have been reported with this class of agents. Due to this risk of secondary malignancy, PARPi have not been evaluated in treating early stage cancer. 

## 3. PARPi as Monotherapy for Metastatic Prostate Cancer 

The landmark phase I study with olaparib reported a >50% reduction in the prostate-specific antigen (PSA) level and resolution of bone metastases after 58 weeks of treatment in a metastatic castration-resistant prostate cancer (mCRPC) patient with germline mutations in *BRCA2* (Table 2) [20]. The dose escalation phase I trial with veliparib included 70 patients with *BRCA2*-mutated mCRPC. The ORR was 37% and among three patients who received the recommended phase II dose of veliparib, their ORR was 66% [21].

The promising responses observed in these phase I studies led to a phase II trial with olaparib in 49 mCRPC patients, who have progressed through standard of care options, including abiraterone, enzalutamide, docetaxel, and cabazitaxel [22]. The primary endpoint was the response rate, defined either as an ORR according to RECIST 1.1, or as a reduction of at least 50% in the PSA level, or a confirmed reduction in the circulating tumor-cell count from five or more cells per 7.5 mL of blood to less than five cells per 7.5 mL. Targeted NGS, exome and transcriptome analysis, and digital polymerase-chain-reaction testing were performed on mandated tumor biopsies. Among the 16 responders, 14 were harbored deleterious genomic alterations in DNA repair genes and most of these genomic alterations were somatic mutations. All seven patients with *BRCA2* loss (four with biallelic somatic loss, and three with germline mutations) and four of five with *ATM* aberrations responded to olaparib. Both patients with either single copy or homozygous deletion of *PALB2* responded to olaparib, whereas two of three patients with homozygous deletion of *FANCA* and one of two patients with homozygous deletion of *CHEK2* responded to olaparib. Based on these data, olaparib was granted breakthrough therapy designation by the FDA for treatment of *BRCA1/2* or *ATM* mutated mCRPC.

TRITON2 is a phase II study that evaluates rucaparib 600 mg twice a day in patients with a deleterious germline or somatic alteration in *BRCA1*, *BRCA2,* or one of 13 other prespecified HRR genes. Patients who progressed on one or two lines of androgen receptor-directed therapy and one prior line of taxane-based chemotherapy for mCRPC are eligible. Prescreening for HRR deficiency with NGS is based on circulating tumor cell (CTC) free DNA and/or tumor biopsy. The primary endpoint that was centrally assessed confirmed ORR per modified RECIST v1.1 for patients with measurable disease and confirmed PSA response (≥50% decrease) in patients without measurable disease. When the initial data were reported at the ESMO 2018, 11 of the 23 patients with *BRCA* mutations had confirmed PSA response (47.8%) [30]. Additionally, 5 of the 11 *BRCA* patients had confirmed radiographic responses (45.5%) and only one patient (1.9%) discontinued rucaparib due to toxicity. Based on these data, FDA granted rucaparib breakthrough therapy designation for *BRCA1/BRCA2*-mutated mCRPC in late 2018. The latest update on this trial was presented at the 2019 American Society of Clinical Oncology (ASCO) annual meeting; among evaluable patients with either germline or somatic deleterious *BRCA1/2* alterations, 44.0% (11/25) had a confirmed radiographic response and 51.1% (23/45) had a confirmed PSA response [31]. Among the 13 preselected HRR genes, most of the responses to rucaparib were observed in patients with *BRCA1* or *BRCA2* mutations. A high concordance rate in detecting mutations in HRR genes was observed between plasma tumor cell free DNA and tumor biopsies.

Niraparib was tested in the phase II GALAHAD study on 39 mCRPC patients with mutations in *BRCA1*, *BRCA2*, *ATM*, *FANCA*, *PALB2*, *CHEK2*, *BRIP1*, or *HDAC2*. The patient population is very similar to TRITON2, i.e., progression on a taxane and at least one androgen-receptor signaling inhibitor in the mCRPC setting. A plasma-based test was developed to screen patients with mutations in these genes. Preliminary results were reported at ASCO Genitourinary Cancers Symposium in early 2019; among 23 patients with *BRCA* mutations, a 38% (5/13) ORR by RECIST, a 57% (13/23) PSA response (decline ≥50%), and 48% (11/23) CTC conversion from above 5 to below 5 were observed [24]. In 16 patients without *BRCA* mutations, the ORR, PSA response, and CTC conversion were 11% (1/9), 6% (1/16), and 31% (5/16), respectively [24]. The toxicity profile is similar to other PARPi with anemia (25%) and thrombocytopenia (15%) being the most common grade 3/4 AEs. 

TALAPRO-1, is a phase II study testing talazoparib in the post-taxane mCRPC population similar to TRITON2. This study had a primary endpoint of ORR with secondary endpoints including time to ORR, DOR, ≥50% decline in PSA, decline of CTC to 0 and <5/7.5 mL blood, time to PSA progression, PFS, and OS. Clinical efficacy and safety data for this trial have not been reported [32].

In summary, preliminary data from phase II PARPi trials have demonstrated more than 50% PSA response rates and around 40% soft tissue response rates in *BRCA1/2*-mutated mCRPC patients who had progression through at least one line of taxane-based chemotherapy and AR signaling inhibitors like abiraterone acetate or enzalutamide. The PARPi activity in non-*BRCA* mutated prostate cancer remains to be proven. Overall PARPi is well tolerated in this post-chemotherapy mCRPC population. Non-hematological grade 3/4 AEs from PARPi treatment were typically below 10%. With transfusion supports and dose adjustments, mCRPC patients rarely discontinue PARPi due to grade 3/4 anemia or thrombocytopenia.

The clinical efficacy and safety of PARPi compared to AR signaling inhibitor in DDR-mutated mCRPC in the pre-chemotherapy setting are being tested in two ongoing phase III trials, PROfound (NCT02975934) and TRITON3 (Table 3). Based on the cross resistance between abiraterone and enzalutamide [33], the investigators hypothesize that PARPi would prolong PFS compare to enzalutamide or abiraterone in patients with DDR-mutated (15 select genes) mCRPC who progressed on an androgen-receptor signaling inhibitor. The PROfound trial with olaparib has completed its enrollment and the enrollment for TRITON3 is ongoing [34]. 

## 4. PARPi Combinations for Treating Metastatic Prostate Cancer

Due to the common side effects of cytopenia, it has been challenging to combine PARPi with cytotoxic chemotherapy. Veliparib and temozolomide is the only reported chemotherapy combination study in mCRPC [26]. Although temozolomide had no single-agent activity against prostate cancer, preclinical cell line and mouse models showed potential synergy between veliparib and temozolomide. This multi-center pilot study did not preselect patients for HRR deficiency. At the time of the report in 2014, only one of 25 evaluable mCRPC patients had a PSA decline of ≥30 (8.0%) [25]. The median PFS and time to progression were only nine weeks [25]. Combining veliparib with temozolomide was well tolerated, with thrombocytopenia (23%) and anemia (15%) being the most common grade 3/4 AEs. Among the PARPi under clinical testing, veliparib is the weakest PARPi in terms of PARP trapping and has the shortest half-life (Table 1). It is the only PARPi that is being combined with cytotoxic chemotherapy in ongoing phase II or III trials in advanced solid tumors.

The recurrent fusions between the androgen-regulated *TMPRSS2* and *ETS* transcription factor genes (primarily *ERG*) occur in about 50% of primary prostate cancers [35]. Preclinical studies showed that PARP1 can interact with *ERG*, and inhibition of PARP1 enhanced DNA double-strand breaks induced by *ERG* overexpression and slowed the growth of *ERG*-positive prostate cancer cells [35]. Further preclinical work demonstrated that PARP1 regulated AR association with chromatin, and controlled AR function. PARP1 inhibition diminished AR activity and sensitized prostate cancer cells to both DNA damage and androgen depletion [36]. Based on these preclinical data, Hussain et al. conducted a phase II randomized study comparing abiraterone and veliparib versus abiraterone alone in 148 patients with mCRPC and these patients were pre-stratified based on the *ETS* fusion status. The study found no difference in PSA response rate for mCRPC patients treated with abiraterone alone versus abiraterone with veliparib (63.9% vs. 72.4%; *p* = 0.27) [26]. Furthermore, no statistically significant difference in ORR and the median PFS were noted between the two treatment arms (ORR, 45.0% vs. 52.2%; *p* = 0.51 and median PFS, 10.1 vs. 11 months; *p* = 0.99). ETS fusion positive mCRPC also lacked benefit from the abiraterone and veliparib combination. Exploratory analysis of this trial did find that mCRPC with DDR mutations had significantly higher PSA responses and longer PFS with the abiraterone and veliparib combination. Additional exploratory analysis revealed that mCRPC with mutations in *PTEN*, *TP53*, and *PIK3CA* had a significantly worse outcome in terms of PFS (*PTEN*, 13.5 vs. 6.7 months, *p* = 0.02; *TP53*, 13.5 vs. 7.7 months, *p* = 0.01; *PIK3CA* 13.8 vs. 8.3 months; *p* = 0.03). 

The role of PARP1 in regulating AR function in prostate cancer and the potential synergy between PARPi and AR signaling inhibitor was tested in another phase II randomized, double-blind study comparing olaparib and abiraterone versus placebo and abiraterone in mCRPC [27]. In this study involving 142 mCRPC patients, olaparib and abiraterone significantly improved radiographic PFS (13.8 vs. 8.2 months; HR 0.65, 95% CI 0.44–0.97 and a *p* = 0.034) with serious AE reported in 24/71 patients (34%) vs. 13/71 (18%) in the control arm. Myocardial infarction was noted in four patients (6%) treated with olaparib and abiraterone versus none in the control arm. Compared to the negative results from the veliparib and abiraterone combination, the improved clinical efficacy and increased toxicity of the olaparib and abiraterone combination is probably attributed to the more potent PARP trapping activity with olaparib compared to veliparib. Based on the efficacy and safety reported in this phase II trial, an ongoing phase III PROpel study (NCT03732820) is testing the abiraterone and olaparib combination versus abiraterone and placebo as a frontline therapy for mCRPC. The primary endpoint of this trial is PFS along with secondary endpoints including time to first subsequent therapy or death, time to pain progression, OS, and health-related quality of life. Of note, neither of these trials requires pre selection for DDR mutations. The effect of DDR mutations on primary endpoint was included in the exploratory analyses.

The phase II randomized, multi-center BRCAAway study (NCT03012321) is another ongoing study testing the combination of PARPi and AR signaling inhibitors as a frontline therapy for mCRPC. Unlike the trials discussed above, this three-arm study preselects patients based on loss of *ATM*, *BRCA1,* or *BRCA2* on tumor biopsies. Patients are randomized 1:1:1 to abiraterone versus olaparib versus abiraterone and olaparib [37].

Talazoparib is also tested in an ongoing phase III trial, TALAPRO-2 (NCT03395197), in combination with enzalutamide compared to enzalutamide alone as a frontline therapy for patients with asymptomatic or mildly symptomatic mCRPC. This trial pre-stratified patients in two cohorts of mCRPC: DDR-mutated and DDR wild-type [38]. The primary endpoint of this trial is PFS in both cohorts along with OS as a secondary endpoint.

The MAGNITUDE study is testing the combination of niraparib with abiraterone compared to abiraterone alone in patients with treatment-naïve mCRPC. This study will stratify patients into two cohorts DDR-mutated and DDR wild-type. The primary endpoint of this trial is PFS in both cohorts along with OS, time to chronic opioid use, time to pain progression, and time to initiation of chemotherapy are the secondary endpoints (NCT03748641).

PARP inhibition has been shown to increase PD-L1 expression in DDR-mutant cancer cells [39]. A recent update of cohort A of Keynote-365 (NCT02861573) reported 32% disease control rate (DCR) of ≥6 months with the olaparib and pembrolizumab combination in post-docetaxel M1 CRPC [28]. Of note, none of the 41 subjects had DDR deficiency. Anemia (27%), neutropenia, fatigue (7%), and asthenia (7%) were the most common grade 3–4 AEs. The potential synergy between PARPi and anti-PD1 or anti-PDL1 therapy was also observed in a small phase II study combining olaparib with durvalumab in M1 CRPC; two of the 17 patients without DDR mutations had a ≥30% decline in PSA [29]. KEYLINK-010 is a phase III study for mCRPC patients who have progressed though one line of AR signaling inhibitor. It tests the hypothesis that the olaparib and pembrolizumab combination will work better than the second line AR signaling inhibitor (NCT03834519). There is no preselection for deleterious mutations in HRR genes required for this trial; PFS and OS are the co-primary endpoints.

In another multicenter, phase III study of pembrolizumab and olaparib, patients with mCRPC and having progressed on an androgen-receptor signal inhibitor are randomized to receive either this combination or abiraterone or enzalutamide.

## 5. Future Directions

The approval of upfront abiraterone for castration-sensitive prostate cancer and the approval of enzalutamide and apalutamide for non-metastatic castration resistant prostate cancer have led to early utilization of potent AR signaling inhibitors in treating advanced prostate cancer. There is an unmet need to develop novel therapies beyond targeting AR signaling for mCRPC. Although PARPi have not been approved for treating prostate cancer yet, both olaparib and rucaparib have FDA-breakthrough designations for *BRCA1/2*-mutated mCRPC. PARPi will likely become the first targeted therapy for HRR-deficient mCRPC. Although all the PARPi under development have shown single-agent activity for *BRCA1/2*-mutated prostate cancer, the efficacy and safety profiles seem to correlate their potency in PARP trapping when PARPi are being combined with abiraterone. Data from ongoing clinical trials will help to address the questions on whether we can apply PARPi beyond *BRCA1/2*-mutated prostate cancer; whether combining PARPi with either AR signaling inhibitors or checkpoint inhibitors will work better in HRR-deficient mCRPC than HRR-intact mCRPC. Other PARPi combinations being tested in clinical trials include combining with radiopharmaceutical, Radium-223 (NCT03317392), and combining with anti-angiogenesis agent, cediranib (NCT02893917) (Table 3). Additionally, employing the use of PARP1 positron emission tomography may help to inform decision making regarding which patients to use PARPi in after stratification based on HRR deficiency or in combination trials [40]. Due to the lack of long-term follow-up data on the safety of PARPi-treated mCRPC and the secondary malignancy (myelodysplastic syndrome, acute myeloid leukemia) observed in PARPi-treated ovarian and breast cancer patients, we would caution testing PARPi or PARPi combinations in early phase prostate cancer.

## Figures and Tables

**Table 1:** Pharmacokinetics of current Poly (ADP-ribose) polymerase inhibitors (PARPi).

Drug (Manufacturer)	Structure	Oral Dosage	Half-Life	Metabolism	PARP Trapping Potency (1–5; 1 = Most Potent)
Olaparib (AstraZeneca)	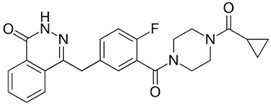	300 mg; twice a day	11.9 h	Hepatic CYP3A4	4
Rucaparib (Clovis Oncology)	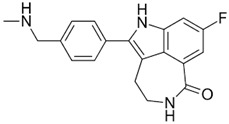	600 mg; twice a day	18 h	Hepatic CYP2D6	3
Niraparib (Tesaro)	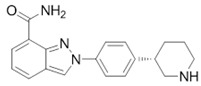	300 mg; daily	36 h	Carboxylesterases	2
Veliparib (AbbVie)	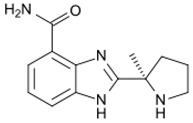	300 mg; twice daily	6.1 h	Hepatic CYP2D6	5
Talazoparib (Pfizer)	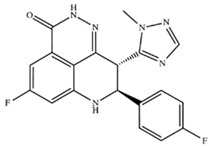	1 mg; daily	90 h	Hepatic mono-oxidation, dehydrogenation, cysteine conjugation of a mono-desfluoro metabolite, and glucuronide conjugation	1

**Table 2:** Completed Phase II PARPi trials in prostate cancer.

Study Name (NCT #)	Patient Population	Sample Size/Number of Pts	Study Design	PSA Response Rate	PFS (If Available)	Dosage	Reference
**Monotherapy**							
TOPARP-B (NCT01682772)	mCRPC progression on abiraterone, enzalutamide, docetaxel, or cabazitaxel	49 (16 with DDR mutations)	olaparib	100% of *BRCA2* and *FANCA* mutated mCRPC drop ≥50% baseline	median PFS, 9.8 vs. 2.7 months; *p* < 0.001	400 mg twice a day	Mateo J et al., [22]
TRITON2 (NCT02952534)	mCRPC and a DDR mutation previously been treated with abiraterone, enzalutamide, docetaxel, or cabazitaxel	52 (23 *BRCA*-mutated)	rucaparib	47.8% of *BRCA*-mutated; 95% CI, 26.8–69.4)	Not reported	600 mg twice a day	Abida W et al., [23]
GALAHAD (NCT02854436)	mCRPC patients with DDR mutations and progression on a taxane or androgen-receptor signaling inhibitor	39	niraparib	57% (95% CI, 34–77)	Not reported	300 mg once a day	Smith MR et al., [24]
**Combination Therapy**							
NCT01085422	mCRPC	26	Veliparib and temozolomide	8.0% (95% CI, 1.0–26.0)	9 weeks (95% CI, 8–17)	40 mg twice a day and 150–200 mg once a day	Hussain M et al., [25]
NCT01576172	mCRPC	148 (76 on abiraterone + veliparib	abiraterone versus abiraterone and veliparib	72.4%	10.1 versus 11 months (*p* = 0.95)	1000 mg once a day and 40 mg twice a day	Hussain M et al., [26]
NCT01972217	mCRPC previously treated with docetaxel or cabazitaxel	142 (71 on the olaparib + abiraterone arm)	abiraterone versus abiraterone and olaparib	Not reported	8.2 versus 13.8 months (*p* = 0.034)	1000 mg once a day and 300 mg twice a day	Clarke N et al., [27]
cohort A of Keynote-365 (NCT02861573)	mCRPC previously treated with docetaxel or ≤2 androgen-receptor signaling inhibitors	41	Pembrolizumab and olaparib	13% of patients had ≥50% PSA decline	5 months (95% CI, 4–8)	200 mg every 21 days and 400 mg twice a day	Yu EY et al., [28]
NCT03810105	mCRPC previously treated with enzalutamide or abiraterone	17	durvalumab and olaparib	53% of patients had a radiographic response and ≥50% PSA decline	16.1 months (95% CI, 4.5–16.1)	1500 mg every 28 days and 300 mg twice a day	Karzai F et al., [29]

**Table 3:** Ongoing PARPi trials in prostate cancer.

Study Name (NCT Number)	Phase	Patient Population	Study Design	Primary Endpoint
**Monotherapy**				
TALAPRO-1 (NCT03148795)	Phase II	DDR-mutated mCRPC progressed on a taxane or androgen-receptor signaling inhibitor	talazoparib	Objective Response Rate
ROAR (NCT03533946)	Phase II	DDR-mutated mCRPC	rucaparib	PSA decline ≥50% rate
Galahad (NCT02854436)	Phase II	DDR-mutated mCRPC who progressed on an androgen-receptor signaling inhibitor and taxane-chemotherapy	niraparib	Objective response rate
TRITON3 (NCT02975934)	Phase III	germline or somatic *BRCA1*, *BRCA2*, or *ATM* mutations and mCRPC who previously progressed on an androgen-receptor signaling inhibitor and who have not received chemotherapy	rucaparib versus abiraterone, enzalutamide, or docetaxel	Progression-free survival
PROfound (NCT02987543)	Phase III	mCRPC who progressed on an androgen-receptor signaling inhibitor	olaparib versus enzalutamide or abiraterone in patients with DDR-mutated	Progression-free survival
**Combination Therapy**				
PROpel (NCT03732820)	Phase III	mCRPC who have not received taxane-chemotherapy or an androgen-receptor signaling inhibitor	abiraterone and olaparib versus abiraterone and placebo	Progression-free survival
BRCAaway (NCT03012321)	Phase II	DDR-mutated mCRPC	Abiraterone versus olaparib and abiraterone versus olaparib	Progression-free survival
TALAPRO-2 (NCT03395197)	Phase III	asymptomatic or mildly symptomatic mCRPC, without brain metastases, never having received taxane-chemotherapy or an androgen-receptor signaling inhibitor	enzalutamide and talazoparib versus enzalutamide and placebo (prestratify based on DDR mutaton status)	Progression-free survival
MAGNITUDE (NCT03748641)	Phase III	treatment naïve mCRPC	niraparib and abiraterone versus abiraterone and placebo	Progression-free survival
KEYLINK-010 (NCT03834519)	Phase III	mCRPC progressed on an androgen-receptor signaling inhibitor	pembrolizumab and olaparib versus enzalutamide or abiraterone	Progression-free survival and Overall survival
NCT03317392	Phase I/Phase II	mCRPC with any number of bone metastases	olaparib and radium-223	Progression-free survival
NCT02893917	Phase II	mCRPC who progressed on one prior line of therapy	olaparib versus olaparib and cediranib	Progression-free survival
NCT03572478	Phase I/Phase IIa	mCRPC who progressed on an androgen-receptor signaling inhibitor	rucaparib and nivolumab	Dose limiting toxicity
NCT03516812	Phase II	mCRPC who progressed on an androgen-receptor signaling inhibitor	olaparib and testosterone injection	PSA decline ≥50% rate
NCT03810105	Phase II	DDR-mutated mCRPC	olaparib and durvalumab	Number of patients with undetectable PSA

## References

[1] Pritchard C.C., Mateo J., Walsh M.F., De Sarkar N., Abida W., Beltran H., Garofalo A., Gulati R., Carreira S., Eeles R. (2016). Inherited DNA-Repair Gene Mutations in Men with Metastatic Prostate Cancer. New Engl. J. Med..

[2] Shaheen M., Allen C., Nickoloff J.A., Hromas R. (2011). Synthetic lethality: Exploiting the addiction of cancer to DNA repair. Blood.

[3] Murai J., Shar-yin N.H., Das B.B., Renaud A., Zhang Y., Doroshow J.H., Ji J., Takeda S., Pommier Y. (2012). Trapping of PARP1 and PARP2 by Clinical PARP Inhibitors. Cancer Res..

[4] Farmer H., McCabe N., Lord C.J., Tutt A.N., Johnson D.A., Richardson T.B., Santarosa M., Dillon K.J., Hickson I., Knights C. (2005). Targeting the DNA repair defect in BRCA mutant cells as a therapeutic strategy. Nature.

[5] Bryant H.E., Schultz N., Thomas H.D., Parker K.M., Flower D., Lopez E., Kyle S., Meuth M., Curtin N.J., Helleday T. (2005). Specific killing of BRCA2-deficient tumours with inhibitors of poly(ADP-ribose) polymerase. Nature.

[6] Weston V.J., Oldreive C.E., Skowronska A., Oscier D.G., Pratt G., Dyer M.J., Smith G., Powell J.E., Rudzki Z., Kearns P. (2010). The PARP inhibitor olaparib induces significant killing of ATM-deficient lymphoid tumor cells in vitro and in vivo. Blood.

[7] McCabe N., Turner N.C., Lord C.J., Kluzek K., Białkowska A., Swift S., Giavara S., O’Connor M.J., Tutt A.N., Zdzienicka M.Z. (2006). Deficiency in the repair of DNA damage by homologous recombination and sensitivity to poly(ADP-ribose) polymerase inhibition. Cancer Res..

[8] Bajrami I., Frankum J.R., Konde A., Miller R.E., Rehman F.L., Brough R., Campbell J., Sims D., Rafiq R., Hooper S. (2014). Genome-wide profiling of genetic synthetic lethality identifies CDK12 as a novel determinant of PARP1/2 inhibitor sensitivity. Cancer Res..

[9] Buisson R., Dion-Côté A.M., Coulombe Y., Launay H., Cai H., Stasiak A.Z., Stasiak A., Xia B., Masson J.Y. (2010). Cooperation of breast cancer proteins PALB2 and piccolo BRCA2 in stimulating homologous recombination. Nat. Struct. Mol. Biol..

[10] Mendes-Pereira A.M., Martin S.A., Brough R., McCarthy A., Taylor J.R., Kim J.S., Waldman T., Lord C.J., Ashworth A. (2009). Synthetic lethal targeting of PTEN mutant cells with PARP inhibitors. EMBO Mol. Med..

[11] Min A., Im S.A., Yoon Y.K., Song S.H., Nam H.J., Hur H.S., Kim H.P., Lee K.H., Han S.W., Oh D.Y. (2013). RAD51C-deficient cancer cells are highly sensitive to the PARP inhibitor olaparib. Mol. Cancer Ther..

[12] Kim G., Ison G., McKee A.E., Zhang H., Tang S., Gwise T., Sridhara R., Lee E., Tzou A., Philip R. (2015). FDA Approval Summary: Olaparib Monotherapy in Patients with Deleterious Germline BRCA-Mutated Advanced Ovarian Cancer Treated with Three or More Lines of Chemotherapy. Clin. Cancer Res..

[13] Ledermann J., Harter P., Gourley C., Friedlander M., Vergote I., Rustin G., Scott C.L., Meier W., Shapira-Frommer R., Safra T. (2014). Olaparib maintenance therapy in patients with platinum-sensitive relapsed serous ovarian cancer: a preplanned retrospective analysis of outcomes by BRCA status in a randomised phase 2 trial. Lancet Oncol..

[14] Coleman R.L., Oza A.M., Lorusso D., Aghajanian C., Oaknin A., Dean A., Colombo N., Weberpals J.I., Clamp A., Scambia G. (2017). Rucaparib maintenance treatment for recurrent ovarian carcinoma after response to platinum therapy (ARIEL3): A randomised, double-blind, placebo-controlled, phase 3 trial. Lancet.

[15] Mirza M.R., Monk B.J., Herrstedt J., Oza A.M., Mahner S., Redondo A., Fabbro M., Ledermann J.A., Lorusso D., Vergote I. (2016). Niraparib Maintenance Therapy in Platinum-Sensitive, Recurrent Ovarian Cancer. New Engl. J. Med..

[16] Friedlander M., Matulonis U., Gourley C., du Bois A., Vergote I., Rustin G., Scott C., Meier W., Shapira-Frommer R., Safra T. (2018). Long-term efficacy, tolerability and overall survival in patients with platinum-sensitive, recurrent high-grade serous ovarian cancer treated with maintenance olaparib capsules following response to chemotherapy. Br. J. Cancer.

[17] Moore K., Colombo N., Scambia G., Kim B.G., Oaknin A., Friedlander M., Lisyanskaya A., Floquet A., Leary A., Sonke G.S. (2018). Maintenance Olaparib in Patients with Newly Diagnosed Advanced Ovarian Cancer. New Engl. J. Med..

[18] Tung N.M., Im S., Senkus-Konefka E., Xu B., Domchek S.M., Masuda N., Li W., Armstrong A.C., Conte P.F., Wu W. (2018). Olaparib versus chemotherapy treatment of physician’s choice in patients with a germline BRCA mutation and HER2-negative metastatic breast cancer (OlympiAD): Efficacy in patients with visceral metastases. J. Clin. Oncol..

[19] Litton J.K., Rugo H.S., Ettl J., Hurvitz S.A., Gonçalves A., Lee K.H., Fehrenbacher L., Yerushalmi R., Mina L.A., Martin M. (2018). Talazoparib in Patients with Advanced Breast Cancer and a Germline BRCA Mutation. New Engl. J. Med..

[20] Fong P.C., Boss D.S., Yap T.A., Tutt A., Wu P., Mergui-Roelvink M., Mortimer P., Swaisland H., Lau A., O’Connor M.J. (2009). Inhibition of poly(ADP-ribose) polymerase in tumors from BRCA mutation carriers. New. Engl. J. Med..

[21] Pahuja S., Appleman L.J., Belani C.P., Chen A., Chu E., Beumer J.H., Puhalla S. (2015). Preliminary activity of veliparib (V) in BRCA2-mutated metastatic castration-resistant prostate cancer (mCRPC). J. Clin. Oncol..

[22] Mateo J., Carreira S., Sandhu S., Miranda S., Mossop H., Perez-Lopez R., Nava Rodrigues D., Robinson D., Omlin A., Tunariu N. (2015). DNA-Repair Defects and Olaparib in Metastatic Prostate Cancer. New Engl. J. Med..

[23] Abida W., Bryce A., Balar A., Chatta G., Dawson N., Guancial E.A., Hussain A., Jha G., Lipsitz D.U., Patnaik A. (2018). TRITON2: An international, multicenter, open-label, phase II study of the PARP inhibitor rucaparib in patients with metastatic castration-resistant prostate cancer (mCRPC) associated with homologous recombination deficiency (HRD). J. Clin. Oncol..

[24] Smith M.R., Sandhu S.K., Kelly W.K., Scher H.I., Efstathiou E., Lara P., Yu E.Y., George D.J., Chi K.N., Summa J. (2019). Phase II study of niraparib in patients with metastatic castration-resistant prostate cancer (mCRPC) and biallelic DNA-repair gene defects (DRD): Preliminary results of GALAHAD. J. Clin. Oncol..

[25] Hussain M., Carducci M.A., Slovin S., Cetnar J., Qian J., McKeegan E.M., Refici-Buhr M., Chyla B., Shepherd S.P., Giranda V.L. (2014). Targeting DNA repair with combination veliparib (ABT-888) and temozolomide in patients with metastatic castration-resistant prostate cancer. Investig. New Drugs.

[26] Hussain M., Daignault-Newton S., Twardowski P.W., Albany C., Stein M.N., Kunju L.P., Siddiqui J., Wu Y.M., Robinson D., Lonigro R.J. (2018). Targeting Androgen Receptor and DNA Repair in Metastatic Castration-Resistant Prostate Cancer: Results From NCI 9012. J. Clin. Oncol..

[27] Clarke N., Wiechno P., Alekseev B., Sala N., Jones R., Kocak I., Chiuri V.E., Jassem J., Fléchon A., Redfern C. (2018). Olaparib combined with abiraterone in patients with metastatic castration-resistant prostate cancer: a randomised, double-blind, placebo-controlled, phase 2 trial. Lancet Oncol..

[28] Yu E.Y., Massard C., Retz M., Tafreshi A., Carles Galceran J., Hammerer P., Fong P.C., Shore N.D., Joshua A., Linch M.D. (2019). Keynote-365 cohort a: Pembrolizumab (pembro) plus olaparib in docetaxel-pretreated patients (pts) with metastatic castrate-resistant prostate cancer (mCRPC). J. Clin. Oncol..

[29] Karzai F., VanderWeele D., Madan R.A., Owens H., Cordes L.M., Hankin A., Couvillon A., Nichols E., Bilusic M., Beshiri M.L. (2018). Activity of durvalumab plus olaparib in metastatic castration-resistant prostate cancer in men with and without DNA damage repair mutations. J. Immunother Cancer.

[30] Abida W., Bryce A.H., Vogelzang N.J., Amato R.J., Percent I., Shapiro J.D., McDermott R., Hussain A., Patnaik A., Petrylak D. (2018). 793PD Preliminary results from TRITON2: A phase II study of rucaparib in patients (pts) with metastatic castration-resistant prostate cancer (mCRPC) associated with homologous recombination repair (HRR) gene alterations. Annals of Oncology.

[31] Abida W., Bryce A.H., Vogelzang N.J., Amato R.J., Percent I., Shapiro J., McDermott R., Hussain A., Patnaik A., Petrylak D. (2019). Genomic characteristics of deleterious BRCA1 and BRCA2 alterations and associations with baseline clinical factors in patients with metastatic castration-resistant prostate cancer (mCRPC) enrolled in TRITON2. J. Clin. Oncol..

[32] De Bono J.S., Higano C.S., Saad F., Miller K., Chen H., Czibere A., Healy C., Fizazi K. (2019). TALAPRO-1: An open-label, response rate phase II study of talazoparib (TALA) in men with DNA damage repair (DDR) defects and metastatic castration-resistant prostate cancer (mCRPC) who previously received taxane-based chemotherapy (CT) and progressed on greater than or equal to one novel hormonal therapy (NHT). J. Clin. Oncol..

[33] Antonarakis E.S., Lu C., Wang H., Luber B., Nakazawa M., Roeser J.C., Chen Y., Mohammad T.A., Chen Y., Fedor H.L. (2014). AR-V7 and resistance to enzalutamide and abiraterone in prostate cancer. New Engl. J. Med..

[34] De Bono J.S., Hussain M., Thiery-Vuillemin A., Mateo J., Sartor A.O., Chi K.N., Fizazi K., Twardowski P., Agarwal N., Sandhu S.K. (2017). PROfound: A randomized Phase III trial evaluating olaparib in patients with metastatic castration-resistant prostate cancer and a deleterious homologous recombination DNA repair aberration. J. Clin. Oncol..

[35] Brenner J.C., Ateeq B., Li Y., Yocum A.K., Cao Q., Asangani I.A., Patel S., Wang X., Liang H., Yu J. (2011). Mechanistic rationale for inhibition of poly(ADP-ribose) polymerase in ETS gene fusion-positive prostate cancer. Cancer Cell.

[36] Schiewer M.J., Goodwin J.F., Han S., Brenner J.C., Augello M.A., Dean J.L., Liu F., Planck J.L., Ravindranathan P., Chinnaiyan A.M. (2012). Dual roles of PARP-1 promote cancer growth and progression. Cancer Discov..

[37] Reichert Z., Carneiro B.A., Daignault-Newton S., Sullivan A., Feng F.Y.C., Morgan T.M., Tomlins S.A., Chinnaiyan A.M., Hussain M. (2017). A randomized phase II trial of abiraterone, olaparib or abiraterone + olaparib in patients with metastatic castration-resistant prostate cancer with DNA repair defects. J. Clin. Oncol..

[38] Agarwal N., Shore N.D., Dunshee C., Karsh L.I., Sullivan B., Di Santo N., Elmeliegy M., Casey M., Quek R.G., Czibere A. (2019). TALAPRO-2: A two-part, placebo-controlled phase III study of talazoparib (TALA) with enzalutamide (ENZA) in metastatic castration-resistant prostate cancer (mCRPC). J. Clin. Oncol..

[39] Chabanon R.M., Muirhead G., Krastev D.B., Adam J., Morel D., Garrido M., Lamb A., Hénon C., Dorvault N., Rouanne M. (2019). PARP inhibition enhances tumor cell-intrinsic immunity in ERCC1-deficient non-small cell lung cancer. J. Clin. Invest..

[40] Makvandi M., Pantel A., Schwartz L., Schubert E., Xu K., Hsieh C.J., Hou C., Kim H., Weng C.C., Winters H. (2018). A PET imaging agent for evaluating PARP-1 expression in ovarian cancer. J. Clin. Invest..

